# Problem-based learning in radiology achieves similar results in classroom and metaverse settings

**DOI:** 10.1186/s13244-025-01987-7

**Published:** 2025-06-12

**Authors:** Teodoro Rudolphi-Solero, Fernando Bajos-Ariza, Rocío Lorenzo-Álvarez, Dolores Domínguez-Pinos, Miguel José Ruiz-Gómez, Francisco Sendra-Portero

**Affiliations:** 1 Department of Radiology and Physical Medicine, Facultad de Medicina—Universidad de Málaga, Bvd. Luis Pasteur, 32, 20071 Málaga, Spain; 2 Primary Care Unit—Centro de Salud Doctor Trueta, Avenida de los Fraguas, 8, 28921 Alcorcón, Spain; 3 Department of Emergency and Intensive Care, Hospital de la Axarquía, Avenida del Sol, 43, 29740 Vélez-Málaga, Spain

**Keywords:** Problem-based learning, Radiology education, Medical students, e-learning, Virtual world

## Abstract

**Objectives:**

The metaverse (MV) is a simulated virtual world enabling simultaneous interaction and communication between students, teachers, and colleagues. This study compared a problem-based learning experience in radiology conducted face-to-face in real life (RL) and within the MV.

**Methods:**

During a radiology clinical rotation, groups of approximately 25 sixth-year medical students participated over 2 years in real life and 2 years in the MV. Each group was divided into eight teams of 3–4 students, each assigned a radiological clinical case for study, presentation, and debate with classmates. Students evaluated other teams, assessed case difficulty, and completed a perception questionnaire.

**Results:**

A total of 348 students participated in the real-life group and 342 in the MV group, with average teacher evaluation scores of 8.11 ± 1.15 and 7.97 ± 1.54, respectively, showing no significant differences (*p* = 0.883). No significant differences were found in peer evaluations or case difficulty assessments. Both groups reported positive experiences, with overall satisfaction scores out of 10 points being 7.91 ± 1.32 for RL and 7.54 ± 1.87 for the MV, without significant differences (*p* = 0.073).

**Conclusions:**

Problem-based learning activities in radiology can be effectively conducted in the MV, yielding academic results and experiential perceptions comparable to RL. The MV presents a viable alternative to face-to-face learning when in-person problem-based learning activities are impractical or challenging.

**Critical relevance statement:**

This study highlights the potential of the metaverse for effectively conducting radiology problem-based learning activities. It provides evidence for its viability as an alternative educational tool, particularly when face-to-face learning is not feasible.

**Key Points:**

- Radiology problem-based learning in the metaverse achieved academic results comparable to traditional real-life classroom settings.
- The metaverse offers unique learning advantages, including remote access, 24/7 availability, and teamwork opportunities.
- The metaverse provides an excellent problem-based learning alternative when in-person activities are impractical or impossible.

**Graphical Abstract:**

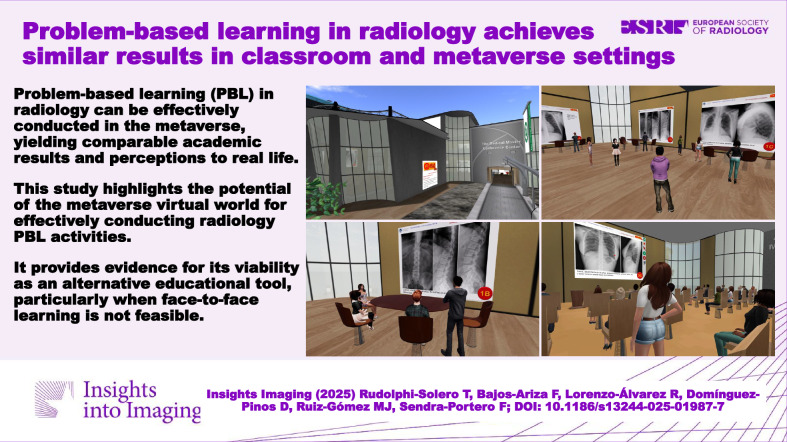

## Introduction

Problem-based learning (PBL), developed by Barrows at McMaster University in the 1960s [1], is a student-centered methodology where students confront a problem, applying prior knowledge and acquiring new insights independently [2]. The teacher shifts from being the focal point to a facilitator guiding the process [3]. This approach enhances high-level cognitive skills and equips medical students with lifelong abilities such as clinical reasoning, problem-solving, and critical decision-making [4]. In Medicine, these “problems” are often presented as clinical cases, leading to a variant known as case-based learning [5]. Substantial evidence supports the effectiveness of PBL in radiology education [6–9], showing its ability to integrate clinical medicine with pathology and anatomy [6], highlight radiology’s relevance in practice [7], tackle diagnostic challenges [8], and enhance engagement, teamwork, and communication skills [9].

Immersive virtual worlds, created through online technology, allow interaction in computer-generated spaces. Along with virtual reality, mirror worlds, and augmented reality, these worlds form the metaverse (MV) [10], a digital universe beyond physical reality. Since 2020, the MV has gained popularity, driven by companies like Meta (formerly Facebook; Meta Platforms, Inc.) and the rise of e-learning during COVID-19 [11]. The MV serves as a learning tool, enabling students to engage with virtual classroom simulations and communicate with peers through avatars, digital representations of users [10].

As background, Second Life (Linden Research, Inc.) is an MV-based virtual environment used to implement the educational intervention in this study. Launched in 2003, it is among the most widely used virtual worlds in health professions education [12, 13]. It allows users to rent and utilize virtual land and supports synchronous (voice or text chat) and asynchronous interactions through timestamped notecards. The platform has been explored in radiology education with medical students [14], for assessing student perceptions [15], and for comparing virtual and in-person settings for radiography seminars [16]. It has shown feasibility for active learning, fostering teamwork and interaction to address radiology challenges [17, 18]. Moreover, PBL experiences using this platform have been conducted in paramedical emergencies [19], psychiatry [20], and nephrology [21], demonstrating its ability to create rich, authentic scenarios. However, to the authors’ knowledge, no PBL experiences in radiology have yet been implemented in this environment.

This study builds on a prior study with sixth-year medical students, showing that PBL practices can be integrated into traditional radiology subjects with high student outcomes, regardless of case difficulty [3]. It hypothesizes that such PBL experiences can be effectively conducted in the MV, achieving similar outcomes and student perceptions. The study compares a PBL radiology experience reproduced identically in face-to-face RL and virtual MV formats, evaluating case difficulty, peer assessment, teacher evaluation, and student perception.

## Material and methods

### Educational context

Sixth-year medical students participated in this study during a 10-day clinical radiology rotation. Seven groups of approximately 25 students each completed the rotation, which included five days of hospital practice alternating with five days of two 2-h radiology seminars on various topics (Table 1). The final seminar on day 9 was reserved for the group meeting for the PBL experience in this study.

**Table 1:** Schedule of the clinical rotation in radiology for ten school days

Day	Activities	
	Seminar (9:30–11:30 h)	Seminar (12:00–14:00 h)
1	Thorax 1	Breast
2	Hospital practices	
3	Thorax 2	Musculoskeletal
4	Hospital practices	
5	Emergency	Abdominal
6	Hospital practices	
7	Pediatric	Central nervous system
8	Hospital practices	
9	Musculoskeletal 2	PBL group meeting^a^
10	Hospital practices	

*PBL* problem-based learning

^a^ During the first 2 years, the PBL group meeting was held face-to-face in RL, the last 2 years it was held online, in the virtual world Second Life

### Development of the learning activity

**Fig. 1:**
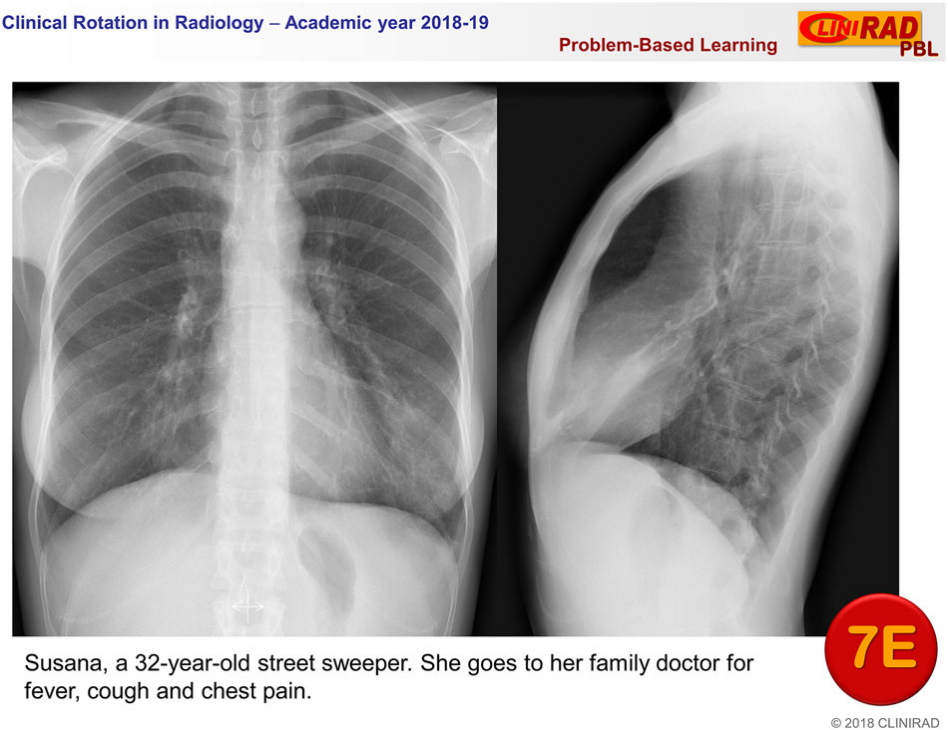
Example of a thoracic case, corresponding to group 7, team E, as it was presented to the students. Radiographs show a subtle alveolar pattern in the lingula, due to pneumonia. The patient names provided were fictitious

On the first day of the rotation, each group was randomly divided into eight teams of 3–4 members. Students received a PDF via the Moodle platform with activity instructions and objectives. Each team was assigned a clinical case consisting of one or two x-rays and a brief description of the patient’s clinical history (Fig. 1). Teams were instructed to:

1. Access the assigned case.
2. Meet with team members.
3. Conduct a systematic analysis of the case.
4. Prepare a response (each team determined the level of detail).
5. Appoint a spokesperson to present the case during the PBL group meeting.

To avoid case repetition, 56 cases were used, including 28 thoracic (50%), 19 musculoskeletal (34%), and 9 abdominal (16%). These cases, prepared by the project’s teachers and used in a prior PBL study [3], represented clinical situations relevant to general medical practice (see Supplementary Material—Appendix A). This study was repeated over four consecutive years, using the same cases with each of the 56 teams to enable result comparison.

**Fig. 2:**
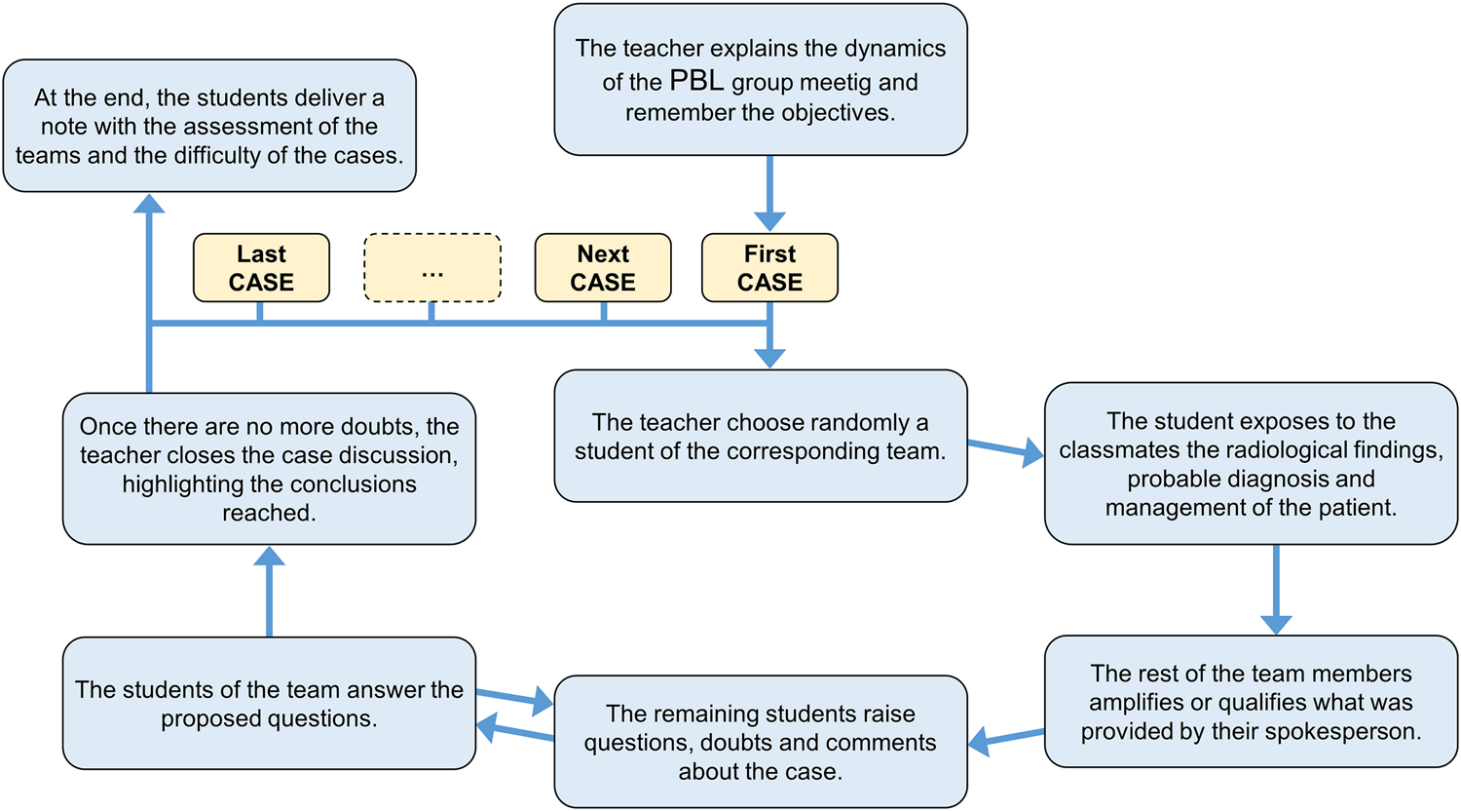
Diagram of case presentation dynamics during the PBL group meeting

The teacher began the 2-h group meeting by outlining the objectives and session dynamics (Fig. 2). Before the presentations, a student from the first team was randomly selected to present their clinical case. Subsequently, the word was given to the remaining team members to clarify or complete the information provided. A brief discussion followed, allowing other students to ask questions, express doubts, or share opinions. Once no further points were raised, the teacher summarized key conclusions without offering an opinion or solution. This process was repeated for all eight teams.

From the beginning of the meeting, students were asked to evaluate the other teams’ work and the difficulty of the cases (including their own) using an evaluation sheet submitted at the end of the meeting.

### Assessment

The teacher evaluated each team using a rubric with six criteria rated from 1 (strongly disagree) to 5 (strongly agree):

1. Systematic reading was correctly performed.
2. Relevant radiological findings were summarized in detail.
3. Radiological findings were adequately correlated with the provided clinical data.
4. Adequate data on patient management were included.
5. An appropriate presentation style was demonstrated.
6. Active participation was observed across the entire team.

The sum of the values obtained in each section was scored from 0 to 10 points using the following formula:

$${Teacher\; evaluation}=\frac{\left({Points}-18\right)\,x\,5}{12}+5$$

Each student evaluated their peers by ranking the top three teams in first, second, and third place (excluding their own team). Teams received one, two, or three points for placing third, second, or first, respectively. The total points were scored out of 10 using the following formula:

$${Peer\; evaluation}=\frac{{Points}}{\left({Sent\; evaluations}-{team\; members}\right)\,x\,3}\,x\,10\,$$

Each student also assessed the difficulty of each case, scoring it from 1 (very easy) to 5 (very difficult).

### The experience in real life (RL) and in the metaverse (MV)

This study spanned four academic years, from 2015–16 to 2018–19. During the first 2 years, PBL activities were conducted in RL. Teams received clinical cases in a PDF via a URL link in the instructions (Fig. 2). Each team decided when and where to meet in person to study their case. Group meetings were held in a seminar room at the medical school, where students submitted paper evaluations before leaving.

**Fig. 3:**
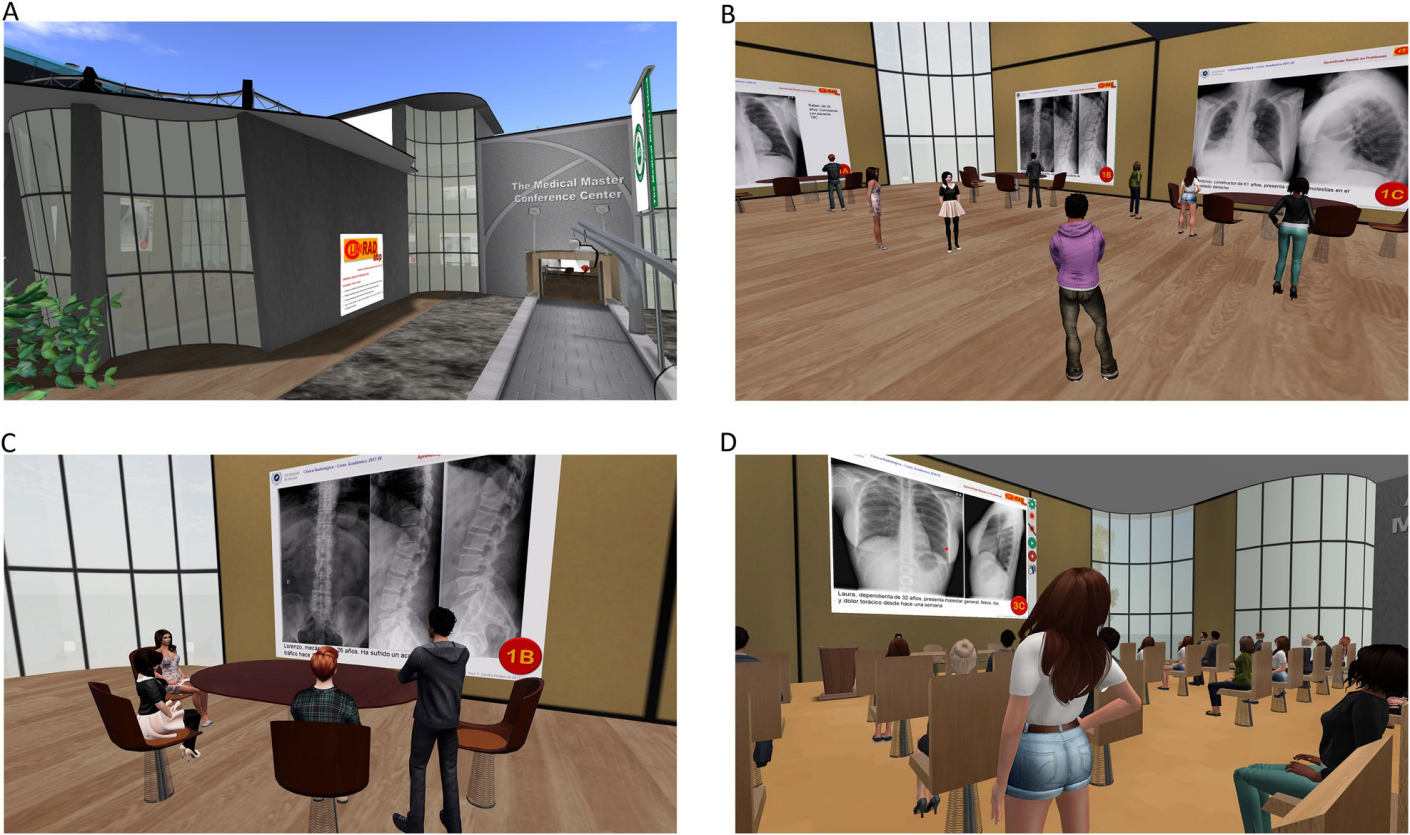
Scenes of Medical Master Island during the PBL experience: Medical Master Conference Center facade (**A**); students in the exhibition hall, analyzing the clinical case assigned to them (**B**); a team discussing the clinical case (**C**); and a randomly selected student explaining their conclusions about a clinical case in a group meeting (**D**)

In the last 2 years, PBL activities were conducted in the Second Life virtual world (Linden Research, Inc.), specifically at “Medical Master Island” [22], a virtual campus where previous radiology education studies were conducted [14–16]. All activities took place in the island’s main building (Fig. 3). Teams accessed their assigned cases on wall panels in an exhibition hall, using the same format as in RL. Students could meet in the hall anytime to discuss their cases. Group meetings were held in a virtual classroom within the same building via voice communication, following the same dynamics as in RL. Students submitted their evaluations using the platform notecard system.

### Perception questionnaire

To assess students’ perceptions, they voluntarily completed a questionnaire used in a previous study [3]. It included a 5-point Likert scale to evaluate 9 statements on the activity’s design, development, and outcomes. Students also answered a dichotomous item on prior PBL knowledge, rated their satisfaction on a scale of 1 to 10, and provided open comments on improvements and observations. In the last 2 years, the questionnaire added a section on the virtual platform, developed in earlier studies [14–16]. This section included 8 statements rated on a 5-point Likert scale, 9 aspects scored up to 10 points, and space for open comments (see Supplementary Material—Appendix B for details).

### Data analysis

All student and questionnaire data were anonymized in compliance with current legislation on personal data protection and digital rights. The study was approved by the ethical experimentation committee of our university (resolution number 001-2023-H, January 25, 2023).

Data were recorded and analyzed using Excel 2013 (Microsoft Cop.) and SPSS version 25 (IBM Corp.). Teacher evaluations, peer evaluations, and case difficulty were compared and correlated using Pearson’s correlation analysis. Additionally, the covariance between teacher and peer evaluations was examined. For the satisfaction questionnaires, means and standard deviations of all assessed aspects were calculated. Open comments were classified using one-layer coding through collaborative systematic coding by group consensus based on their content [23], with each comment tagged into multiple codes as applicable.

## Results

This study included 690 students, distributed over four consecutive years as 162, 186, 168, and 181 students, with 348 in the RL group and 342 in the MV group. Of these, 669 (97.0%) completed the peer evaluation and case difficulty assessment, while 607 (88.0%) submitted the perception questionnaire.

**Fig. 4:**
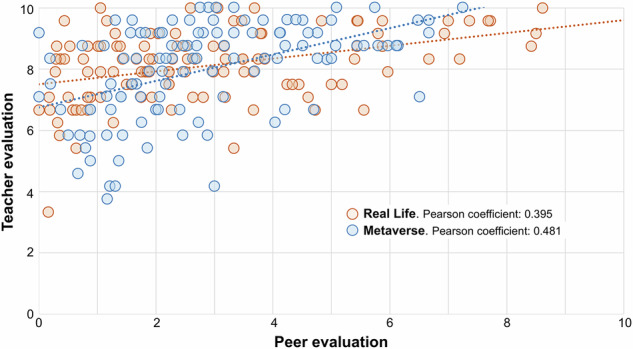
Scatter diagram that correlates the score obtained by each team in the peer evaluation and in the teacher evaluation in the real life and second life subgroups. The Pearson correlation coefficient for the 4 years of the study was 0.424 (*p* = 0.01); covariance = 1.129

A comparison between case difficulty and peer evaluation revealed no significant correlation (Pearson’s correlation = 0.02; *p* = 0.761). However, case difficulty and teacher evaluation showed a weak negative correlation (Pearson’s correlation = −0.195; *p* = 0.03), while peer and teacher evaluations demonstrated a moderate correlation (Pearson’s correlation = 0.424; *p* = 0.01). The covariance study yielded a positive value of 1.129, indicating a correlation between the highest values (Fig. 4). No significant differences were found between RL and MV for the variables (Table 2). Average difficulty scores were 3.34 ± 0.59 for thorax cases, 3.10 ± 0.56 for musculoskeletal cases, and 2.82 ± 0.72 for abdomen cases, with significant differences among thematic groups. However, RL and MV groups showed no significant differences in difficulty scores for any case type.

**Table 2:** Comparison of evaluations and difficulty between face-to-face and virtual environments

Variables	Real life	Metaverse	Mann–Whitney *U* (*p*)
Peer assessments: *N* (Percentage)	340 (97.7%)	329 (96.2%)	
Peer evaluation^a^	2.86 ± 2.17	2.86 ± 1.72	*0.494*
Case difficulty^b^	3.18 ± 0.69	3.17 ± 0.57	*0.717*
Teacher evaluation^a^	8.11 ± 1.15	7.97 ± 1.54	*0.883*

Values in italics indicate statistically significant differences comparing both gropus (*p* < 0.05)

^a^ Teacher evaluation and peer evaluation were normalized to 10 points

^b^ Case difficulty was scored up to 5 points

Of the 607 completed questionnaires, 298 were from the RL group (85.6%) and 309 from the MV group (90.4%). Table 3 shows the PBL evaluation questionnaire results comparing both groups. Responses on the 5-point Likert scale had mean values ranging from 3.79 to 4.41, with significant differences in only three of the nine assessments. Overall satisfaction averaged 7.71 out of 10, with no significant differences between both the groups. Prior PBL knowledge was reported by 86 students (29.1%) in the RL group and 229 students (74.1%) in the MV group.

**Table 3:** Students’ perception of the PBL experience comparing the real life and metaverse groups.

Statements	Real life		Metaverse		*p*^a^	Total	
	Mean ± std	Median (IC)	Mean ± std	Median (IC)		Mean ± std	Median (IC)
The group meeting was adjusted to the objectives of the subject.	4.37 ± 0.74	4 (4.29–4.45)	4.24 ± 0.85	4 (4.14–4.34)	0.169	4.30 ± 0.79	4 (4.24–4.37)
The information prior to the meeting was appropriate.	3.80 ± 0.90	4 (3.70–3.90)	4.04 ± 0.96	4 (3.94–4.15)	< 0.001	3.92 ± 0.94	4 (3.85–4.00)
The design of the content provided was adequate.	4.13 ± 0.77	4 (4.05–4.22)	4.07 ± 0.99	4 (3.97–4.17)	0.794	4.10 ± 0.85	4 (4.03–4.17)
The methodology of the experience was correct.	4.04 ± 0.85	4 (3.94–4.14)	3.93 ± 1.07	4 (3.81–4.05)	0.644	3.99 ± 0.97	4 (3.91–4.06)
The cases were adjusted to the needs of the subject.	4.13 ± 0.84	4 (4.04–4.23)	4.18 ± 0.90	4 (4.08–4.28)	0.307	4.16 ± 0.88	4 (4.09–4.23)
The organization of the experience was correct.	4.25 ± 0.86	4 (4.15–4.34)	4.27 ± 0.91	5 (4.16–4.37)	0.519	4.26 ± 0.88	4 (4.19–4.33)
The selection of the presenter for each team was appropriate.	4.06 ± 1.07	4 (3.94–4.18)	4.41 ± 0.86	5 (4.32–4.51)	< 0.001	4.24 ± 0.99	4 (4.16–4.32)
The experience was useful for my needs.	4.24 ± 0.90	4 (4.13–4.34)	3.79 ± 1.16	4 (3.66–3.92)	< 0.001	4.01 ± 1.06	4 (3.92–4.09)
The experience met my expectations.	3.95 ± 0.93	4 (3.95–4.03)	3.88 ± 1.14	4 (3.76–4.01)	0.944	3.92 ± 1.04	4 (3.83–4.00)
My overall satisfaction with the experience was.^b^	7.91 ± 1.32	8 (7.76–8.06)	7.54 ± 1.87	8 (7.33–7.74)	0.073	7.72 ± 1.04	8 (7.59–7.85)

^a^ The test performed was the Mann–Whitney’s *U*-test

^b^ This statement was rated out of 10 points. The rest of them responded in a 5-point Likert scale from 1 (strongly disagree) to 5 (strongly agree)

**Fig. 5:**
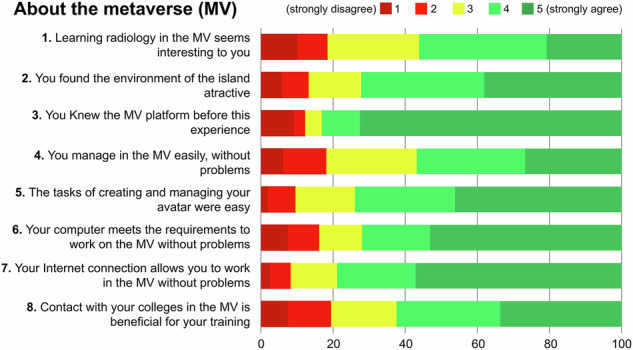
Students’ perception about the virtual platform experience answering statements in a 5-point Likert scale

**Fig. 6:**
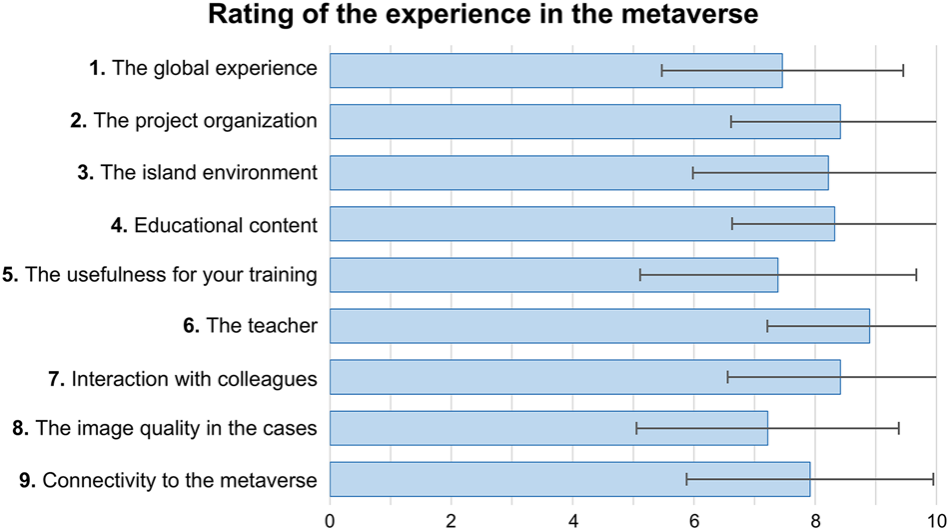
Students’ perception of the virtual platform experiences, rating nine items up to 10 points

Regarding the MV, most students agreed (rating 4 and 5) that they were familiar with the platform beforehand (83.1%), had sufficient Internet or computer access (78.9% and 71.3%, respectively), found avatar management easy (73.9%), and considered the island environment attractive (72.2%). Disagreement (ratings 1 and 2) with the eight statements was below 20% (Fig. 5). Various aspects of the MV platform received average scores above 7.3 out of 10, with the teacher, project organization, educational content, and classmate interaction rated highest, averaging over 8.2 (Fig. 6).

Three hundred seventy-eight students (62.3%) provided open comments: 183 (61.4%) from the RL group and 195 (63.1%) from the MV group. Only comments on the PBL experience or the MV platform were analyzed; others related to radiology or the clinical rotation were excluded. Table 4 presents thematic coding of comments about the PBL experience, categorized into suggestions for improvement, positive comments, and less frequent topics. The most common suggestion (54.5%) in both the RL and MV groups was for the teacher to provide the answers to the cases. Ten percent suggested extending PBL to include more cases, modalities, or subjects. Positive feedback was abundant, with 33.1% expressing appreciation and 23.3% highlighting the educational value of the experience. Other comments (6–7%) mentioned teamwork, innovation, relevance to practice, and gratitude toward teachers.

**Table 4:** Thematic coding of open comments about the PBL experience and frequency of appearance in the Real life (RL) and metaverse (MV) groups

CODE	Meaning			Frequency		
				RL	MV	Total
Suggestions for improvement						
Final resolution	The students requested that the professor provide them with the final solution to the cases, alleging various reasons and proposing various solutions		119		87	206
More PBL	They propose that the PBL methodology be extended further in the degree, increasing the number of cases and sessions, or including other modalities of radiology or other subjects.		18		20	38
More clinical data	They suggest that cases be accompanied by more clinical data or a more detailed clinical history.		7		8	15
Presenter	Disagreement with the random choice of the presenter in the group meeting, thinking it harms the team’s outcome		13		2	15
Difficulty	Pointing out the heterogeneity in the difficulty of the cases and/or suggesting greater homogeneity in this aspect.		5		1	6
Positive comments						
Appreciation	Expressing that they liked the experience, with words such as good, timely, positive, attractive, interesting, even terms like magnificent or exceptional.		45		80	125
Useful	Speaking of the educational usefulness of the experience, with terms such as productive, formative, didactic, appropriate, useful for learning		35		54	88
Teamwork	Highlighting the relationship between colleagues and the importance of teamwork in the experience		10		18	28
Innovative	Referring to innovation, with synonymous terms such as different, original, new or surprising.		5		23	28
Actual practice	They relate the experience to the clinical activity or nearby postgraduate professional practice.		17		8	25
Acknowledgement	Words of recognition or gratitude to teachers for the work done.		11		13	24
Fun	The experience was fun, pleasant, entertaining, or enjoyable.		5		12	17
Interaction	Highlight the interaction, discussion, and transmission of knowledge between colleagues in the entire group.		4		13	17
Motivating	The experience involves them, motivates them, commits them, or is rewarding for them.		5		3	8
Comments on less frequent themes						
Previous skills	They acknowledge having a previous low level in the interpretation of radiographs.		1		4	5
Time lack	They consider that they have too many academic activities and a lack of time to dedicate to additional learning activities.		0		4	4
More time	They consider that they need more time to be able to meet and resolve the cases.		4		0	4
Team choice	They propose that students freely choose team members.		4		0	4
Summative exams	They indicate that the contents must be oriented towards the final summative exams.		1		2	3
PBL orientation	They suggest prior training on PBL methodology for resolving cases.		1		1	2
Others	Various comments on isolated topics		4		3	7

Of the 195 open comments from the MV group, 133 (68.2%) addressed the virtual platform and were grouped into positive comments, suggestions, and limitations or technical issues (Table 5). The most frequent comments expressed appreciation for the MV platform (26.3%) or a preference for face-to-face meetings (24.1%).

**Table 5:** Thematic coding of open comments about the metaverse and frequency of appearance

CODE	Meaning		Frequency
Positive comments			
Appreciation	Expressing words of appreciation for the MV platform in this experience, that they liked it, or that they found it good, adequate, successful, interesting, or positive.		35
Remote access	Highlighting the advantage of connecting remotely, from home.		16
More MV	Suggesting that the use of the MV platform be extended to other seminars, other subjects, or new teaching projects.		13
Fun	Describing that using Second Life for learning is fun, enjoyable, or entertaining to them.		7
Persistence	Highlighting the advantage of accessing the platform whenever and however much you want to review the content.		4
Participation	Highlighting that the MV encourages participation, interaction, and debate, even more than in RL.		3
Suggestions or preferences			
Real life	Expressing a preference for in-person face-to-face experiences.		32
Time schedule	Dissatisfaction with the afternoon schedule established for group sessions, and/or suggesting a morning schedule.		8
Download images	Requesting that the case images be downloadable.		8
School access	Recommending that the Faculty of Medicine’s Wi-Fi connection be enabled, or that computers be available to use SL.		6
MV Training	Requesting training with the platform at the beginning of the rotation.		5
Other platforms	Suggesting other online platforms other than the MV one to carry out this experience.		3
Record	Suggesting video recording the sessions so that they can be reviewed later.		1
Limitations and technical issues			
Image quality	Describing insufficient quality in some radiological images.		15
Platform	Describing that they do not like the MV platform, or that they find it uncomfortable or unintuitive.		14
Distraction	Indicating that the management of the platform makes it difficult to follow the session and communicate fluently.		11
Internet	Referring to Internet connection problems that do not allow them to use SL correctly.		11
Audio	Reporting having had audio communication problems, their own or those of other colleagues, during the session.		10
Computer	Referring to problems with the computer that prevent them from using SL correctly.		8
Avatar camera	Referring to problems with the management of the camera (zoom, angulation of the avatar’s vision) to see the images properly.		8

*MV* metaverse

## Discussion

In this study, a PBL experience in radiology was implemented with final-year medical students in both traditional classrooms and the MV, maintaining the same structure, organization, and content. Academic outcomes were comparable, and student perceptions were similar. While the MV cannot replace face-to-face communication due to the lack of non-verbal cues, it is a valuable alternative when physical presence is unfeasible, as seen during COVID-19 restrictions [24, 25], or for remote collaboration between students and teachers [26].

Previous studies show that the MV is as effective as traditional lecture-based methods for teaching radiotherapy content [27] and that radiography interpretation seminars can be successfully replicated in 3D virtual classrooms with outcomes comparable to face-to-face settings [16]. This study confirms similar results, demonstrating that the entire PBL process, including case preparation, teamwork, plenary discussions, and evaluations, can be conducted in the MV. Additionally, in this environment, 3D imaging modalities such as CT, MRI, or PET/CT can be incorporated through stack presentation, as is being implemented in other educational projects pending publication.

PBL is recommended in modern teaching for improving student performance and knowledge acquisition in health education [5]. It also enhances long-term learning and social skills [28], essential for medical practice. Consistent with earlier research [3], this study found that PBL outcomes were independent of case difficulty, yielding high satisfaction and good learning results in both RL and MV settings.

A moderate positive correlation was observed between teacher and peer evaluations, aligning with other studies showing modest correlations between peer and traditional evaluations in clinical clerkships [29]. However, in this study, the evaluation systems differed, as students only ranked the top three groups, while professors evaluated all groups. Greater alignment in evaluation criteria could improve correlations.

In the final 2 years of the study, more students were familiar with the PBL methodology, reflecting progress in updating teaching approaches in our Faculty of Medicine. This may also explain higher agreement on organizational aspects, such as pre-meeting information and team presenter selection.

The high number of open comments reflects the commitment students showed in both cohorts, offering numerous suggestions for improvement and abundant positive feedback. Over half suggested that teachers provide final case resolutions, with some perceiving the lack of feedback as a learning gap. In traditional clinical seminars, where professors provide solutions, students may adopt a passive approach, exerting minimal effort [3]. When a final solution is absent, students may experience uncertainty and even anxiety about whether their approach was correct. These negative feelings are commonly linked to uncertainty during clinical rotations [30] and social anxiety [31], underscoring the need for a Pavlovian learning model where feedback—positive or negative—is crucial [32]. Uncertainty is an inherent part of medical practice and an uncomfortable reality for many doctors [33, 34]. Poorly handled uncertainty can lead to higher healthcare costs and a more paternalistic approach [35]. In PBL, problem-solving should be driven by peer discussion, with the degree of certainty guiding success [3]. Medical students must learn to manage uncertainty in clinical settings, a skill that teamwork helps develop [36]. Academic training addressing uncertainty is vital for medical practice and should be included in medical school curricula [37, 38].

Research highlights the value of exploring virtual environments such as the MV in medical education [12, 39]. Although it has been used in radiology teaching [14–18, 26, 40], its application to radiology PBL remains underexplored. The MV can replicate face-to-face activities and offers benefits like remote access, 24/7 availability, anonymity, opportunities to develop communication skills, and cost-free participation [12, 14, 39]. Consistent with previous studies [16, 39], our findings show that PBL can be effectively delivered in both virtual and in-person formats without compromising academic performance. The MV also supports real-time discussion, with avatar anonymity helping to reduce social anxiety and discomfort with public speaking [16].

Students in the RL cohort found the PBL experience more useful than those in the MV cohort. In-person experiences foster stronger connections with activities and are more familiar to students than virtual environments, which require adaptation. The Technology Acceptance Model explains this disparity, as studies during the COVID-19 pandemic suggest that for students to fully embrace mandatory virtual teaching, they must perceive it as useful, easy to use, and enjoyable [41, 42].

The opinion of the MV group regarding the virtual platform was generally very positive. The teacher, project organization, educational content, and peer interaction were rated highest, with scores exceeding 8.2 out of 10. The platform was well accepted in technical aspects like computer requirements, internet connectivity, and avatar management, likely due to prior experience (83%) [18, 40]. One of the items with the lowest agreement was “Learning radiology in the MV seems interesting to you.” This may be explained by two factors also reflected in open comments: first, not all students are motivated to adopt new technologies like the MV [41]; second, mandatory online activities from home may not be well received, even if conducted in a recreational context [18]. Some students questioned the need for a virtual seminar when it could be held in person. However, MV-based PBL is particularly valuable when in-person activities are not feasible, such as in distance education, postgraduate training, or inter-university collaborations. This study highlights the strengths and limitations of both virtual and face-to-face environments. While MV platforms may involve recurring costs—such as virtual land rental, server access, or platform subscriptions—these vary by provider and customization level. In contrast, face-to-face teaching typically relies on existing university infrastructure, often incurring no additional costs. Thus, while virtual environments offer new opportunities, their implementation may require additional resources that should be carefully considered. This study did not assess cost-effectiveness.

This study has several limitations. Differences in teaching and peer evaluation methodologies may have introduced comparison bias. Specifically, student scores were based on the ranking of the top three teams in each group, which could have influenced results. Additionally, the study compared cohorts from different academic years rather than using randomized participation, which may have introduced bias, such as greater PBL familiarity among MV students. Long-term academic outcomes and direct comparisons between final exams and PBL performance were not assessed. Another limitation relates to the technical requirements of the MV platform: 16.2% and 8.3% of participants reported inadequate computer resources or internet access, consistent with previous studies [14–18]. This may impact the feasibility of implementation in low-resource settings or among students from disadvantaged backgrounds. To mitigate this, some students suggested providing Wi-Fi access or MV-compatible computers within the Faculty of Medicine.

## Conclusion

PBL activities in radiology, adapted to a conventional course structure, can be effectively conducted in the MV, achieving comparable academic results and similar student perceptions to those in RL. MV technology offers several advantages, including remote access, cost-effective implementation, 24/7 availability, and opportunities for active learning and public speaking practice. However, while the MV provides a viable alternative when in-person PBL activities are impractical, it does not fully replace the benefits of real-life interaction. In this study, some students showed a preference for RL learning environments over the MV, suggesting that virtual platforms should be considered a complementary rather than a primary learning tool.

## Supplementary information

ELECTRONIC SUPPLEMENTARY MATERIAL

## Data Availability

The datasets used and/or analyzed during the current study are available from the corresponding author on reasonable request.

## Abbreviations

COVID-19: Coronavirus Disease 2019
MV: Metaverse
PBL: Problem-based Learning
RL: Real life
