# Supplementary material for: Problem-based learning in radiology achieves similar results in classroom and metaverse settings
Source: Insights Imaging. 2025 Jun 12;16:121. doi: 10.1186/s13244-025-01987-7 (PMC12162394; doi:10.1186/s13244-025-01987-7)
Supplement: Supplementary file 1 — ELECTRONIC SUPPLEMENTARY MATERIAL [file 13244_2025_1987_MOESM1_ESM.pdf]

Problem-Based Learning in Radiology achieves similar results in classroom and metaverse settings.

ELECTRONIC SUPPLEMENTARY MATERIAL

Appendix A. Description of the cases used in this study and assessment of the difficulty by the students.

Case	Description	Radiograph/s	Findings	Difficulty *
1	35-year-old man. Living with a patient with tuberculosis.	Chest, PA	Subtle alveolar infiltrate in the right upper lobe.	3.36±0.89
2	26-year-old man mechanic. He has suffered a traffic accident 12 days ago.	Thoracolumbar spine, AP and lateral	Crush fracture of the L1 vertebral body.	2.35±0.86
3	61-year-old man, builder. He consulted for dyspnea and discomfort on the right side of the thorax.	Chest, PA and lateral	Middle lobe atelectasis and right pleural effusion.	3.99±0.86
4	67-year-old retired man. He went to the emergency room for significant abdominal pain.	Abdominal, AP	Significant pneumoperitoneum.	2.65±0.92
5	64-year-old man, waiter, and owner of a cafeteria. He comes to the consultation for chest pain for 30 days that is increasing. He says that he "suffers from the bronchi".	Chest, PA and lateral	Ill-defined mass in the right lower lobe. Right lung hypoventilation and linear atelectasis.	3.79±0.82
6	18-year-old male student. He has suffered from Crohn's for 4 years. Currently he refers to back pain that is becoming more intense.	Thoracolumbar spine, AP and lateral	Osteopenia and collapse of the lumbar vertebral endplates.	3.24±0.90
7	59-year-old woman. History of recent cardiac surgery	Chest, PA and lateral	Median sternotomy sutures, metallic mitral and aortic valve prostheses, transient automatic defibrillator and mild bilateral pleural effusion.	3.06±0.82
8	17-year-old female student. She has severe abdominal pain and fever.	Abdominal, AP	Mass that occupies the pelvis, causing colon obstruction.	3.14±1.00
9	72-year-old male retired farmer, who presented chronic	Chest, PA and lateral	Bilateral, diffuse reticular interstitial	3.85±0.78

	dyspnea and fever of recent onset and poor general condition.		pattern, with a non-circumscribed alveolar pattern in the apical segment of the right upper lobe.	
10	4.5-year-old boy. The mother takes him to the doctor because she has noticed that he limps.	Pelvis, AP and hip abduction	Deformity and reduction of the epiphysis of the left femoral head. Poorly placed gonadal protector.	2.70±0.86
11	A 55-year-old male office worker with a previous surgical history. He went to the emergency room for chest pain and tachycardia.	Chest, PA	Median sternotomy sutures. Aortic valve prosthesis. Double left cardiovascular contour indicative of descending aortic aneurysm.	3.99±1.08
12	7-year-old boy. His mother insists that he has swallowed a coin	Abdominal, AP	Round, metallic foreign body located in the pyloric antrum.	1.95±0.90
13	A 62-year-old man, construction foreman, smoker of 1.5 packs of cigarettes a day, reports a feeling of shortness of breath and tiredness "for a long time".	Chest, PA	Marked pulmonary emphysema with flattening of diaphragms and peripheral linear pattern.	3.10±0.79
14	20-year-old woman. She has been suffering from diarrhea and occasional abdominal pain for a year. In a matter of two days she has developed a picture of pain and abdominal distension.	Abdominal, AP	Dilation of the cecum, ascending and transverse colon, with loss of haustra and identification of pseudopolyps in its wall. Sudden obstruction in the splenic angle.	2.79±0.83
15	29-year-old woman, housewife. She went to the emergency room with a fever of 39º and dyspnea.	Chest, PA	Bilateral micronodular miliary pattern with thickening of the right paratracheal line and occupation of the aortopulmonary recess.	3.49±0.91
16	45-year-old woman, administrative. She presents bilateral knee pain.	Knees, bilateral AP weight-bearing	Marked osteopenia and diffuse narrowing of both joint spaces. Deformity of both tibial plateaus and signs of osteoarthritis in the left medial compartment.	3.14±0.83
17	51-year-old man, mechanic. He went to his Primary Care Center for chest pain of 15 days of evolution.	Chest, PA	Subtle nodule in the right lung and osteolytic metastasis in the right seventh costal arch.	3.93±0.81

18	38-year-old man, economist. He went to the emergency room due to intense abdominal and lumbar pain, radiating to the pelvis, which woke him up, causing him to vomit.	Abdominal, AP	Left kidney stone superimposed on the last rib.	2.11±0.93
19	32-year-old shop assistant. She has had a general malaise, fever, cough and chest pain for a week.	Chest, PA and lateral	Segmental alveolar pattern in the right upper lobe, with an air-fluid level indicating cavitation.	3.15±0.92
20	A 55-year-old man presents to the emergency room with significant pain and abdominal distension.	Abdominal, AP	Marked dilation of a loop of the sigmoid colon, with a "coffee bean" sign indicative of volvulus. No evidence of perforation.	2.77±1.14
21	74-year-old woman. An evolutionary follow-up X-ray has been performed due to known previous pathology.	Chest, PA	Right mastectomy. Widening of the upper mediastinum with deviation of the trachea to the right and presence of a calcified nodule, indicative of intrathoracic goiter. Calcified liver cyst.	3.33±0.88
22	18-year-old student who goes to his health center for cervicobrachialgia that has been going on for several months.	Cervical spine, lateral	Fusion of vertebral bodies and spinous processes of C5-C6 that modifies cervical lordosis.	2.59±0.87
23	62-year-old cancer patient who comes to the emergency room due to dyspnea.	Chest, PA	Medical devices: subcutaneous reservoir with tip in superior cava, tracheostomy cannula and nasogastric tube. Left cervical mass. Moderate right pneumothorax.	2.95±0.78
24	10-year-old boy. He is brought to the health center due to trauma to his left thigh while he was running through the park.	Left femur, AP and lateral.	Distal diaphyseal fracture, with anteromedial angulation, on a lytic lesion with well-defined and sclerotic edges.	2.63±0.94
25	A 42-year-old office worker goes to his family doctor for dyspnea that has been going on for several months, which has recently become more intense.	Chest, PA	Increased cardiothoracic index, bilateral hilar enlargement with vascular morphology. Peripheral Kerley B lines.	3.26±1.10

26	24-year-old man, with a wrist fracture 2 weeks ago. He comes because “the cast on his elbow bothers him and it hurts”.	Right elbow, AP and lateral	Depressed fracture of the anterior portion of the radial head.	3.29±0.97
27	70-year-old man, smoker and chronic bronchopath. He comes for worsening dyspnea and hemoptysis.	Chest, PA	Bilateral thick reticular interstitial pattern, widening of the right upper mediastinum and rounded opacity with a lobulated contour in the right lung.	4.09±0.87
28	Housewife, 66 years old. Cholecystectomy 12 years ago, hysterectomy 6 years ago. Pain when walking for a month that is causing limp	Pelvis, AP	Fissure in the left ileopubic ramus and osteolytic lesion in the left ischium.	3.88±0.81
29	43-year-old nurse. Control x-rays.	Chest, PA andlateral	Subcutaneous reservoir with tip in superior cava. Left breast expander with metal valve in the anterior region.	2.45±0.81
30	42-year-old bank employee. He goes to the emergency room, sweaty, with significant pain and abdominal distension.	Abdominal, AP	Dilation of loops of small intestine and colon with marked presence of pneumoperitoneum.	3.84±0.81
31	49-year-old waiter currently unemployed. He goes to his health center for dyspnea.	Chest, PA	Atelectasis of the right upper lobe with the "Golden S" sign.	2.65±0.79
32	53-year-old butcher. He has pain in his left foot that prevents him from walking.	Left foot, dorsoplantar	Erosion of the medial margin of the first metatarsal head with swelling of the adjacent soft tissues.	2.70±0.83
33	A 70-year-old retired man undergoes a preoperative study for inguinal hernia surgery.	Chest, PA	Discrete pulmonary emphysema. Subtle right retrodiaphragmatic pulmonary nodule	3.52±0.88
34	A 60-year-old housewife has been experiencing right-sided abdominal pain for months.	Abdominal, AP	Increase in size of the gallbladder that rejects the intestinal loops, with five radiopaque gallstones.	3.20±1.05
35	56-year-old woman, teacher, undergoing an x-ray check.	Chest, PA and lateral	Right mastectomy. Nodular thickening of the right paratracheal line and occupation of the right hilum indicative of lymphadenopathy.	3.42±0.99
36	A 27-year-old woman presents with occasional low-grade	Abdominal, AP	Displacement of intestinal loops	2.96±0.90

	fevers and diffuse abdominal pain for weeks, which is increasing.		suggesting hepatomegaly. Other findings: Metallic artifacts from clothing (bra) and phleboliths.	
37	48-year-old woman, office worker. She undergoes an x-ray as part of a health checkup for a work stay in Australia.	Chest, PA	Cavitation in the right lung with thin, well-defined walls, suggestive of pneumatocele.	3.03±0.96
38	Retired truck driver, 59-years-old, diabetic, has inflammation and a small ulcer on his right foot.	Right foot, dorsoplantar and oblique.	Complete lysis of the head of the fifth metatarsal and partially of the base of the proximal phalanx, with tumor of adjacent soft tissues.	3.34±1.00
39	46-year-old woman, economist. No clinical data.	Chest, PA and lateral	Left breast expander with metal valve in the lateral region.	2.37±0.99
40	56-year-old professor who presents pain and functional limitation in his left shoulder.	Left shoulder, AP in neutral position and internally rotated.	Calcification in the supraspinatus tendon, visible in the image with arm rotation.	2.96±1.06
41	36-year-old delivery man. He has had a cold for two weeks that is not improving. He has been out for two days, with a fever of 38.5º, chills and chest pain.	Chest, PA and lateral	Subtle alveolar pattern, retrocardiac, in the left lower lobe.	2.78±0.71
42	A 9-year-old boy reported pain in the upper area of his left leg for two weeks, after a contusion while playing soccer.	Left femorotibial region, AP and lateral.	Lytic lesion in the proximal peroneal diaphysis, centralmedullary, well defined, which inflates the cortex without destroying it.	3.06±0.81
43	75-year-old housewife. A post-surgical control x-ray is performed 18 months after the last intervention.	Chest, PA and lateral	Global cardiomegaly with signs of left atrial dilation.	3.28±0.90
44	25-year-old student comes to consultation for headache and photophobia	Nasosinusal region, Waters view	Agenesis of frontal sinuses. Bilateral scalloped thickening of the walls of the maxillary sinuses without signs of bone destruction.	3.60±1.03
45	48-year-old man under study for dyspnea and poor general condition.	Chest, PA	Subtle bilateral interstitial pattern with bilateral hilar thickening	3.59±0.72

			and widening of the right paratracheal line.	
46	Housewife, 66 years old. Cholecystectomy 12 years ago, hysterectomy 6 years ago. Shoulder pain on mobilization.	Both shoulders, AP	Ill-defined, lytic lesion on the medial margin of the head of the right humerus.	4.10±0.76
47	22-year-old student. Preoperative study.	Chest, PA	Dextroposition of the aortic arch.	3.06±1.06
48	68-year-old retired man. He presents generalized bone pain and asthenia.	Skull, lateral	Multiple rounded lytic lesions in the skull, suggestive of multiple myeloma.	2.84±0.86
49	A 65-year-old housewife with a history of surgery and progressive dyspnea that has recently worsened.	Chest, PA	Left mastectomy. Bilateral reticular pattern with mass in the right pulmonary apex.	3.89±0.82
50	36-year-old delivery man. Traffic accident.	Cervical spine, lateral	Longitudinal fracture of the vertebral body C4 with retrolisthesis on C5. Fractures of the posterior arch of C4, and spinous process of C3.	3.15±0.90
51	A 32-year-old woman, a lawyer, presents with low-grade fevers for three weeks. Occasional cough, she does not show other relevant symptoms.	Chest, PA	Left mediastinal widening in the aortopulmonary recess that obliterates the aortic arch.	3.14±0.88
52	49-year-old woman, dressmaker. She has cervicobrachialgia that has been going on for years and has become more intense lately.	Cervical Spine, lateral	Rectification of cervical lordosis, with signs of discovertebral osteoarthritis in C3-C4 and C4-C5.	2.81±0.85
53	32-year-old woman, sweeper. She goes to her family doctor for fever, cough, and chest pain.	Chest, PA and lateral	Segmental alveolar infiltrate in the lingula.	2.92±0.84
54	49-year-old woman, administrative, who consults for bilateral back pain.	Knees, bilateral AP weight-bearing	Calcification of the menisci (chondrocalcinosis). Narrowing of the right medial compartment with signs of osteoarthritis.	2.97±0.87
55	30-year-old man, welder. Admitted for dyspnea.	Chest, PA	Subtle right subcutaneous emphysema. Right	3.70±0.96

			pneumothorax difficult to appreciate.	
56	31-year-old man, plumber. He has persistent pain in his right wrist after suffering a fall while playing soccer.	Right wrist, dynamic dorsopalmar.	Transverse scaphoid fracture.	3.26±0.82

*Difficulty data are presented as mean ± standard deviation from a 5-point scale from 1 (very easy) to 5 (very difficult). Number of observations per case ranged from 65 to 71.

Appendix B. Example of the perception questionnaire on PBL.

Please take a few minutes to complete this anonymous questionnaire. Group number ☐

(1 strongly disagree - 5 Strongly agree)

	1	2	3	4	5
**DESIGN**					
The group meeting was adjusted to the objectives of the subject	☐	☐	☐	☐	☐
The information prior to the meeting was appropriate	☐	☐	☐	☐	☐
The design of the content provided was appropriate	☐	☐	☐	☐	☐
**DEVELOPMENT**					
The methodology of the experience was correct	☐	☐	☐	☐	☐
The cases were adjusted to the needs of the subject	☐	☐	☐	☐	☐
The organization of the experience was correct	☐	☐	☐	☐	☐
The selection of the presenter for each team was appropriate	☐	☐	☐	☐	☐
**RESULT**					
The experience was useful for my needs	☐	☐	☐	☐	☐
The experience met my expectations	☐	☐	☐	☐	☐

I previously knew about problem-based learning (Yes/No) ☐

My overall satisfaction with the experience was **(respond up to 10 points)** ☐

IMPROVABLE ASPECTS AND OTHER COMMENTS

Appendix B. Example of the perception questionnaire on Second Life.

Please complete the following questions by checking the box, rating from 1 to 5 (1: strongly disagree, 5: strongly agree)

About Second Life	1	2	3	4	5
Learning Radiology in Second Life seems interesting to you	☐	☐	☐	☐	☐
You found the environment of the island attractive	☐	☐	☐	☐	☐
You knew Second Life before this experience	☐	☐	☐	☐	☐
You manage in Second Life easily, without problems	☐	☐	☐	☐	☐
The tasks of creating and managing your avatar were easy	☐	☐	☐	☐	☐
Your computer meets the requirements to work on Second Life without problems	☐	☐	☐	☐	☐
Your Internet connection allows you to work in Second Life without problems	☐	☐	☐	☐	☐
Contact with your colleagues in Second Life is beneficial for your training	☐	☐	☐	☐	☐

Important! Rating up to 10

*Finally, please rate the following aspects in the boxes, **scoring up to 10 points**.*

The global experience	☐
The project organization	☐
The island environment	☐
Educational content	☐
The usefulness for your training	☐
The teacher	☐
Interaction with colleagues	☐
The image quality in the cases	☐
Connectivity to Second Life	☐

Free comments on Second Life

If you want to add something, please use the box below
